# Potential Molecular Associations Between Triphenyl Phosphate Exposure and Thyroid Cancer: Integration of Network Toxicology and Machine Learning for Core Target Identification with Molecular Docking

**DOI:** 10.3390/ijms27136018

**Published:** 2026-07-04

**Authors:** Yongling Pei, Junxi Liu, Zixin Liu, Meng Xiao, Bohou Xia, Yamei Li

**Affiliations:** 1 Key Laboratory for Quality Evaluation of Bulk Herbs of Hunan Province, School of Pharmacy, Hunan University of Chinese Medicine, Changsha 410208, China; 202304020216@stu.hnucm.edu.cn (Y.P.); 202304020211@stu.hnucm.edu.cn (J.L.); 202502010817@stu.hnucm.edu.cn (Z.L.); 20243756@stu.hnucm.edu.cn (M.X.); xiabohou@hnucm.edu.cn (B.X.); 2 Hunan Engineering Technology Research Center for Rapid Test and Removal of Adverse Substances in Traditional Chinese Medicine, School of Pharmacy, Hunan University of Chinese Medicine, Changsha 410208, China

**Keywords:** triphenyl phosphate, thyroid cancer, network toxicology, machine learning, molecular docking, hub genes

## Abstract

Triphenyl phosphate (TPhP) is a ubiquitous environmental contaminant and endocrine disruptor potentially associated with an increased risk of thyroid cancer (TC). However, whether TPhP directly contributes to TC remains unclear. This study integrated network toxicology and machine learning to investigate potential molecular associations between TPhP exposure and thyroid oncogenesis. By integrating multi-source databases and transcriptomic data, we constructed a TPhP–TC interaction network and established a TC risk prediction model using 127 machine learning algorithm combinations, identifying ten candidate hub genes. GO and KEGG enrichment analyses indicated that these genes are predominantly enriched in phosphorus metabolism, purine metabolism, and nuclear receptor signaling pathways, implying that TPhP may be linked to tumorigenesis through the disruption of metabolic reprogramming. SHAP analysis highlighted AHR and SLC20A2 as critical contributors to model performance. Molecular docking predicted stable binding between TPhP and all hub proteins in silico, with binding energies ranging from −9.2 to −6.6 kcal/mol. This study offers two computational contributions: (1) a quantifiable framework for predicting pollutant-associated TC risk and (2) systematic computational evidence for potential TPhP thyroid toxicity. These findings address a critical gap in understanding potential links between endocrine-disrupting chemical exposure and thyroid carcinogenesis, generating hypotheses for future experimental validation.

## 1. Introduction

Thyroid cancer (TC) is the most prevalent malignant tumor of the endocrine system, with its global incidence continuously rising, representing a burgeoning public health concern [1]. According to GLOBOCAN 2022 data, approximately 821,000 new TC cases were reported worldwide, ranking fifth among malignancies in females [2]. Notably, thyroid cancer is the most common endocrine malignancy, with a particularly high burden in women and a steadily increasing incidence in both sexes over recent decades [3]. Projections indicate that by 2034, the incidence of TC in China may reach 1.62 million, more than doubling within a single decade, while fatalities are predicted to rise from 14,400 to 32,600 [4]. The complex etiological landscape of TC encompasses genetic susceptibility, ionizing radiation, and exposure to environmental chemicals, among which environmental endocrine-disrupting chemicals (EDCs) are recognized as pivotal drivers of TC development [5].

Triphenyl phosphate (TPhP), an organophosphate flame retardant and plasticizer, is extensively utilized as a replacement for polybrominated diphenyl ethers in electronics, furniture, building materials, and consumer products [6]. Since TPhP is physically incorporated rather than chemically bonded within these materials, it exhibits a high susceptibility to leaching into the environment during the process of production, utilization, and disposal. Consequently, TPhP has been detected globally in water bodies, sediments, indoor dust, and biota [7,8,9]. Humans are persistently exposed to TPhP through inhalation, dietary intake, and dermal contact [10]. Notably, TPhP and its metabolites are frequently detected in human urine, serum, breast milk, and placental tissues, highlighting its status as a ubiquitous environmental contaminant [11]. The recent upsurge in the consumption of takeaway food has further escalated human exposure, as TPhP can migrate from plastic packaging under high-temperature or high-fat conditions [12,13].

Existing studies have documented the toxic effects of TPhP on various biological systems. However, whether exposure to TPhP directly contributes to the initiation and progression of TC, as well as its underlying molecular mechanisms, remain inadequately understood. The pathogenesis of TC involves a complex interaction among genetic mutations, dysregulation of signaling pathways, and alterations in the tumor microenvironment. Environmental chemicals may promote tumorigenesis via multi-target and multi-pathway interactions [14]. Traditional toxicological research commonly focuses on individual targets, which restricts its ability to comprehensively depict the holistic relationship between complex environmental exposures and disease conditions. Network toxicology, an emerging discipline integrating computational toxicology and systems biology, enables a systematic analysis of the intricate molecular networks between pollutants and diseases [15]. By combining target prediction, protein–protein interaction (PPI) networks, and functional enrichment analyses, this approach effectively identifies critical molecular events. Furthermore, the integration of machine learning and SHAP (SHapley Additive exPlanations) interpretability techniques has recently enhanced the predictive precision and explanatory power of toxicological modeling [16].

Based on this background, the present study investigates whether TPhP exposure may be associated with TC progression through the modulation of specific hub target genes and their associated signaling networks. To explore this hypothesis, we adopted a network toxicology methodology. Multi-source databases were integrated to predict human targets of TPhP, and transcriptomic data were utilized to identify TC-associated differentially expressed genes (DEGs). Intersection analysis was performed to identify TPhP–TC interaction targets, followed by the application of 127 machine learning algorithm combinations and SHAP analysis to prioritize hub genes. Finally, molecular docking was employed to predict the direct interactions between TPhP and hub proteins. The present study seeks to systematically elucidate the molecular associations between TPhP exposure and thyroid cancer, providing novel computational insights into TPhP-associated carcinogenesis and offering theoretical support for environmental risk assessment and regulatory policies.

## 2. Results

### 2.1. Toxicity Characteristics of TPhP

The typical two-dimensional structure of TPhP was extracted from the PubChem database. SMILES: C1=CC=C(C=C1)OP(=O)(OC2=CC=CC=C2)OC3=CC=CC=C3.

The toxicity assessment of TPhP employed two predictive toxicology platforms: ADMETLAB 3.0 and ProTox-3 (Supplementary Materials S1 and S2). The two tools showed partial agreement but also notable discrepancies (Table 1). Notably, all predicted toxicities should be considered as computational hypotheses and require experimental validation.

### 2.2. Putative Targets of TPhP

**Figure 1:**
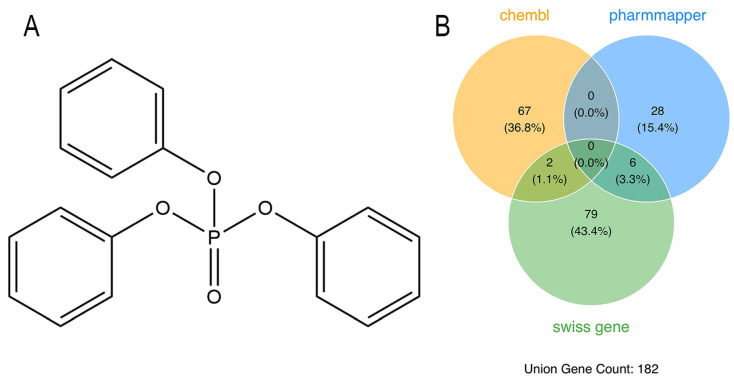
Acquisition of TPhP target proteins. (**A**) Chemical structure of TPhP. (**B**) TPhP-related gene set.

We retrieved the molecular structure of TPhP from the PubChem database (Figure 1A).

Potential human target genes associated with TPhP were systematically searched through three databases: ChEMBL, SwissTargetPrediction, and PharmMapper. Initial searches yielded 69 target genes from ChEMBL, 87 target genes from SwissTargetPrediction, and 34 target genes from PharmMapper. Integration and deduplication of these datasets resulted in a final count of 182 unique genes, defined in this study as the TPhP-related gene set. The overlap distribution of genes among databases is illustrated in Figure 1B.

### 2.3. TC-Associated Genes Identified by Integrated Analysis

#### 2.3.1. Identification of Differentially Expressed Genes in TC

**Figure 2:**
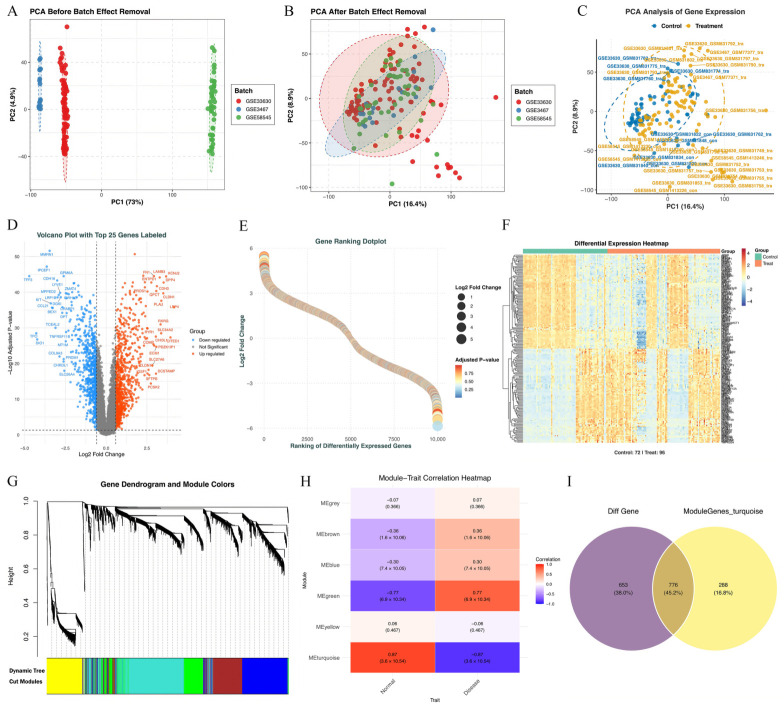
Identification of thyroid cancer-related target genes. (**A**) PCA plot before batch effect removal. (**B**) PCA plot after batch correction. (**C**) Differential gene expression PCA plot. (**D**) Volcano plot of DEGs based on logFC and significance. (**E**) Circular plot of overall distribution of differential genes. (**F**) Heatmap showing DEG expression patterns among samples. (**G**) Gene dendrogram plot from WGCNA. (**H**) Module–trait relationship heatmap. (**I**) Venn diagram, DEG module (purple) and WGCNA module (yellow).

To minimize batch effects, we merged the GES33630, GSE3467, and GSE58545 datasets and comprehensively normalized the gene expression matrices. Principal Component Analysis (PCA) showed improved data distribution post-normalization. Before normalization, the three datasets were distinctly separated (Figure 2A), whereas post-normalization, the datasets exhibited a clearer clustering pattern (Figure 2B), indicating a reduction in batch effects and facilitating subsequent biological differential analysis.

#### 2.3.2. Expression Patterns of TC-Associated DEGs

To investigate the impact of cancer status on the normal tissue transcriptome, differential expression analysis was conducted following successful batch effect removal. We revealed systematic differences in the transcriptome profiles between the cancer tissues and normal tissues, which constituted the primary source of sample variation (Figure 2C). Furthermore, we observed a significant number of upregulated genes in the cancer tissues, with expression levels markedly higher than those in the normal tissues and high statistical significance, indicating a strong transcriptional response (Figure 2D,E). Similarly, numerous downregulated genes in the cancer tissues had significantly lower expression than in the normal tissues, with similar significance and number to the upregulated genes. We demonstrated that the screened differential genes (including both upregulated and downregulated genes) could clearly distinguish the normal tissues from the cancer tissues (Figure 2F), as evidenced by the distinct color patterns.

#### 2.3.3. Gene Co-Expression Modules Associated with TC

To ensure that the gene co-expression network conformed to the scale-free topology commonly seen in biological networks, we constructed a systematic co-expression model using WGCNA. Initially, the network topology structure was systematically evaluated through a soft-threshold power function, implementing a grid search over candidate power values ranging from 1 to 20 and calculating the corresponding scale-free topology model fit index R2. The minimum value satisfying the scale-free topology criterion (R2 ≥ 0.8) was *β* = 5. Based on the selected *β* value, the topological overlap matrix (TOM) was calculated among genes. Genes were clustered using a hierarchical clustering algorithm based on TOM distance and combined with dynamic tree cutting strategies; genes with similar expression patterns were grouped into the same module. By adjusting the minimum number of genes per module and module merging thresholds (deepSplit parameter), eight biologically significant co-expression modules were ultimately identified. For facilitation of visualization and subsequent tracking, each module was color-coded (Figure 2G). After module identification, module–trait association analysis was performed by conducting Pearson correlation analysis between module characteristic genes and TC clinical traits, revealing a significant correlation between specific modules and TC (*p* < 0.05) (Figure 2H). This module is considered a core module closely related to TC pathogenesis and will be the focus of subsequent hub gene screening and functional enrichment analysis.

#### 2.3.4. Integration of TC-Related Genes from Multiple Sources

By intersecting the differentially expressed genes with the WGCNA module genes and removing duplicates, we ultimately identified 776 candidate genes closely associated with TC pathogenesis (Figure 2I). These genes were used in subsequent analyses.

### 2.4. Overlapping Targets Between TPhP and TC

#### 2.4.1. Identification of Intersection Genes with TPhP Targets

**Figure 3:**
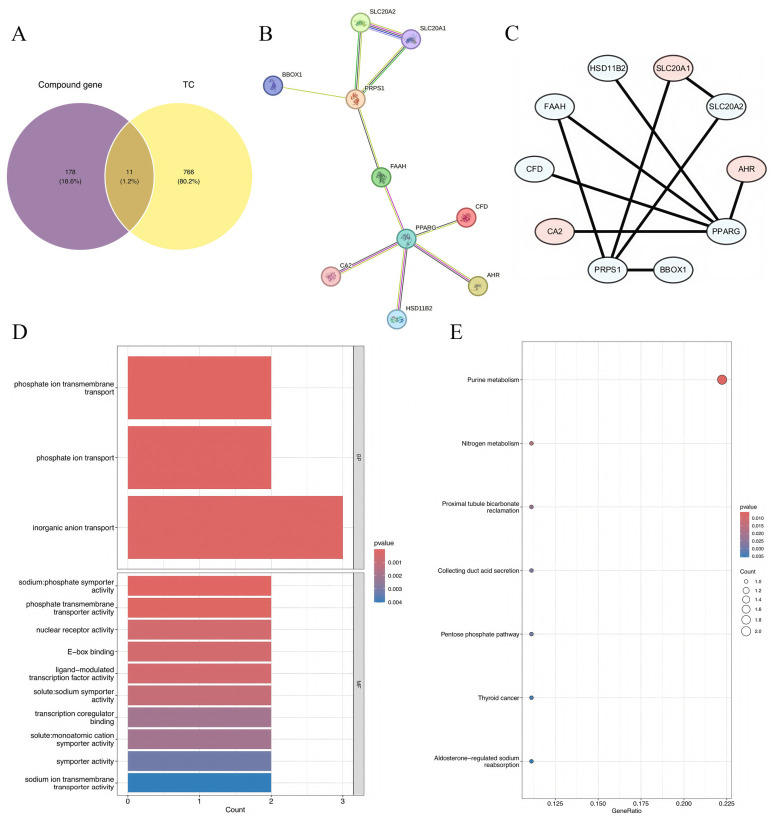
Identification of TPhP-related disease targets in thyroid cancer. (**A**) Venn diagram showing 11 overlapping genes (1.2%) between TPhP (purple) and TC (yellow). (**B**) PPI network of TPhP–TC core genes. (**C**) Visualization of interactions among overlapping genes in the PPI network. Red nodes = upregulated; blue nodes = downregulated; edges = predicted interactions. (**D**) GO enrichment annotations for overlapping genes in BP and MF (no significant CC entries were shown (*p* ≥ 0.05), so they are not displayed). (**E**) KEGG analysis showing enriched pathways of gene overlap.

Intersecting the 776 TC-associated genes with the 182 TPhP-predicted targets yielded 11 candidate genes: CFD, PRPS1, AHR, SLC20A2, FAAH, PDE10A, PPARG, HSD11B2, BBOX1, SLC20A1, and CA2 (Figure 3A). These genes are computationally predicted to be associated with both TPhP and thyroid cancer.

#### 2.4.2. Sensitivity Analysis of DEG Thresholds

To test whether the identification of the 11 core genes depended on the chosen DEG cutoffs, we repeated the intersection procedure using lenient (|log_2_FC| > 0.3, FDR < 0.1) and stringent (|log_2_FC| > 1.0, FDR < 0.01) thresholds, while keeping the turquoise module genes and TPhP targets fixed. Under the lenient threshold, all 11 genes were recovered (triple intersect = 12). Under the original threshold (|log_2_FC| > 0.585, FDR < 0.05), the 11 genes were also fully recovered (triple intersect = 11). Under the stringent threshold, 5 of the 11 genes (CFD, AHR, HSD11B2, BBOX1, SLC20A1) remained (triple intersect = 5). These results indicate that the core candidate set is robust to moderate changes in the DEG thresholds, whereas very stringent filtering inevitably reduces sensitivity (Supplementary Materials S3).

#### 2.4.3. Protein–Protein Interaction (PPI) Network

We submitted the 11 candidate genes to the STRING database for PPI analysis using a confidence threshold of ≥0.7. This analysis retained 10 genes with strong interactions; one gene was excluded as an isolated node (Figure 3B). We visualized the resulting PPI network using Cytoscape (v3.10.3) (Figure 3C). Thus, 10 core genes were identified as associated with the TPhP–TC interaction.

### 2.5. Functional Enrichment of TPhP–TC Core Genes

We performed GO and KEGG enrichment analyses to annotate the functions of the identified targets and gain computational molecular insights. The GO enrichment results (Figure 3D) show that several items have very small *p*-values (all ≤0.004), suggesting statistical significance and a potential focus on phosphate metabolism and transport. The most significantly enriched Biological Process (BP) entries include phosphate ion transmembrane transport and inorganic anion transport, involving key genes such as SLC20A2 and SLC20A1. In the Molecular Function (MF) category, enrichment is high for sodium–phosphate cotransporter activity, phosphate transmembrane transporter activity, nuclear receptor activity, E-box binding, and ligand-regulated transcription factor activity, involving regulatory genes like AHR and PPARG. Additionally, significant enrichment is observed for metabolism-related functions such as carbonic anhydrase activity, cyclic nucleotide phosphodiesterase activity, fatty acid binding, and steroid dehydrogenase activity.

KEGG pathway enrichment analysis (Figure 3E) reveals the gene set primarily enriched in metabolic pathways, such as purine metabolism, nitrogen metabolism, and the pentose phosphate pathway, alongside pathways related to the excretory system, like bicarbonate reabsorption in proximal tubules and aldosterone-regulated sodium reabsorption, as well as thyroid cancer pathways. Enriched genes include CA2, PRPS1, PPARG, and HSD11B2.

The two analyses are consistent with each other: ion transport and metabolic enzyme functions enriched in GO correlate with participation in purine/nitrogen metabolism and excretory system pathways in KEGG; nuclear receptor transcription regulatory activities enriched in GO (AHR, PPARG) relate to cancer pathways, including thyroid cancer, in KEGG. Furthermore, it is noteworthy that phosphate ion transmembrane transport (GO) is mechanistically linked to purine metabolism (KEGG) because intracellular phosphate availability directly regulates the rate-limiting enzyme PRPS1, which controls purine nucleotide synthesis. Dysregulation of phosphate homeostasis may thus lead to aberrant purine metabolism, fueling nucleotide demand for rapid cell proliferation, a hallmark of tumorigenesis. Conversely, targeting phosphate transport or purine metabolic pathways has been explored as a therapeutic strategy in various cancers. In the context of thyroid cancer, the coordinated enrichment of phosphate transport and purine metabolism suggests a potential metabolic vulnerability that could be exploited for prevention or treatment, although experimental validation is required. Overall, these computational results suggest that the target gene set may play synergistic roles in related biological processes by regulating inorganic ion homeostasis, intervening in nucleotide and energy metabolism, and participating in nuclear receptor-mediated signaling.

### 2.6. Core Targets Screened by Machine Learning

Internal cross-validation in the discovery cohort: Among the 127 model combinations evaluated by 10-fold cross-validation, we selected the Lasso + Stepglm [both] model as the final model based on its favorable cross-validation performance, with a mean cross-validation AUC of 0.982 (range: 0.909–1.000, SD = 0.03). The ten individual fold AUCs were 0.986, 0.909, 0.958, 1.000, 1.000, 1.000, 1.000, 0.971, 1.000, and 1.000, indicating stable performance without severe overfitting.

**Figure 4:**
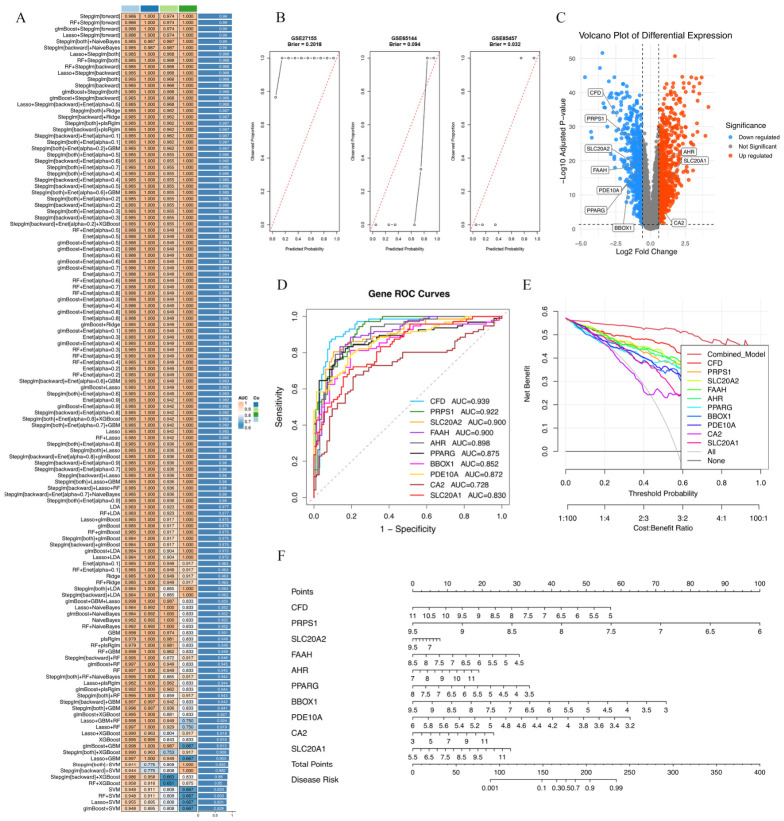
Machine learning analysis. (**A**) Heatmap displaying AUC values of different models across various cohorts, used to compare model performance. Left column = models; right column = AUC. (**B**) Calibration curves of the final model on three independent validation datasets (GSE27155, GSE65144, GSE85457). The *X*-axis shows the predicted probability and the *Y*-axis shows the observed proportion of events. The diagonal dashed line represents perfect calibration. Brier scores are indicated in each panel. (**C**) Volcano plot of differentially expressed genes (DEGs), with key genes marked. The *X*-axis shows log fold change (logFC) and the *Y*-axis shows −log10 (*p*-value). Red indicates upregulated genes, while blue indicates downregulated genes. (**D**) ROC curves for the key genes, illustrating their individual predictive capabilities. (**E**) Decision curve analysis (DCA) plot showing the net benefit of the combination gene model and various strategies over a range of threshold probabilities. (**F**) Nomogram plot for predicting individual disease risk based on gene expression levels. The plot provides a visual representation of the contribution of each gene, where total scores correspond to a range of disease risk probabilities.

External validation on three independent datasets: The final locked model was evaluated separately on GSE27155, GSE65144, and GSE85457; the resulting performance metrics are summarized in Table 2. Calibration curves (Figure 4B) showed good agreement between predicted and observed probabilities, with Brier scores of 0.202, 0.094, and 0.032, respectively.

Based on the selected model, ten core genes were identified: CFD, PRPS1, AHR, SLC20A1, SLC20A2, FAAH, PDE10A, PPARG, BBOX1, and CA2. Their differential expression in thyroid cancer tissues was visualized using a volcano plot (Figure 4C), where upregulated genes are marked in red and downregulated genes in blue. The diagnostic efficacy of these key genes was further examined through ROC curve analysis (Figure 4D). Decision curve analysis (DCA) (Figure 4E) revealed that across a wide range of threshold probabilities, the net benefit of the combination gene model was higher than that of single-gene models and the “all intervention” strategy, suggesting a potential advantage in clinical applications. A nomogram plot (Figure 4F) enables individual disease risk prediction, with each gene’s expression level corresponding to a specific score. The total score aligns with a disease risk range from 0.001 to 0.99, with varying contributions from different genes. Variables such as CFD and PRPS1 contributed larger proportions to the total score.

### 2.7. SHAP-Based Feature Importance Analysis

**Figure 5:**
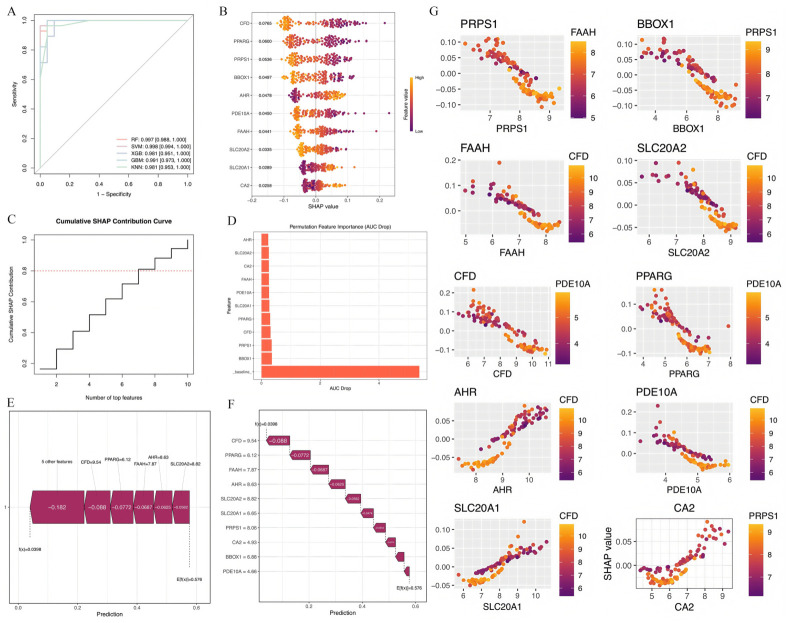
Machine learning model performance and SHAP analysis of core genes. (**A**) ROC curves distinguishing disease groups using different machine learning models. Shows the receiver operating characteristic curves and area under the curve (AUC), with 95% confidence intervals for six models: Random Forest (RF), Support Vector Machine (SVM), XGBoost (XGB), Gradient Boosting Machine (GBM), and K-Nearest Neighbor (KNN). (**B**) Beeswarm plot of SHAP values for the top ten genes. Each dot represents the SHAP value of a specific feature in a single sample, with color indicating feature expression level (red for high expression, blue for low expression). Features are ordered from top to bottom by average absolute SHAP value. (**C**) Cumulative SHAP contribution curve. Shows the cumulative contribution proportions of the top-ranked features to the model’s total interpretability, sorted by average absolute SHAP value. (**D**) Feature importance based on AUC decline after permutation. The bar chart shows the extent of AUC reduction after each feature is shuffled, reflecting the impact of each gene on model performance. (**E**) SHAP force plot. Presents the direction and magnitude of each feature’s contribution to the prediction for a single sample; red indicates positive contributions (pushing the prediction towards the disease group), blue indicates negative contributions (pushing the prediction towards the control group). (**F**) SHAP waterfall plot for a representative sample. Displays the process of individual features contributing cumulatively from the baseline expected value (E [f(x)]) to the final prediction value (f(x)), visually showing the cumulative impact on the individual prediction. (**G**) SHAP dependency plots for core genes. Each plot shows the relationship between feature expression level (*X*-axis) and SHAP value (*Y*-axis), revealing the direction, strength, and nonlinear contribution patterns of features to the model’s predictions across different samples.

We constructed multiple machine learning classification models based on the expression levels of the 10 core genes. ROC analysis showed that all evaluated models had excellent discriminative performance (Figure 5A), indicating that the selected gene signature effectively distinguished disease samples from control samples.

SHAP analysis further revealed the contribution patterns of each core gene to model predictions. The SHAP summary plot (Figure 5B) identifies CFD (SHAP value range approximately −0.08 to +0.08), PPARG, and PRPS1 as the most influential predictors. Notably, these genes exhibit bidirectional effects on model output: high expression (shown in red) predominantly aligns with negative SHAP values, pushing predictions toward the control group, while low expression (shown in blue) aligns with positive SHAP values, driving predictions toward the disease group.

The cumulative contribution curve (Figure 5C) reveals that the top three genes contribute to over 40% of the model’s explanatory power, the top six genes collectively contribute more than 70%, and the top eight genes cumulatively account for nearly 90%. This result highlights that the model’s predictions rely heavily on a small set of key features, consistent with the focused nature of the core gene selection.

Feature importance ranking using permutation (Figure 5D) shows that AHR and SLC20A2 produce the most significant reduction in AUC after feature shuffling, indicating their critical importance for model performance.

The SHAP force plot (Figure 5E) and waterfall plot (Figure 5F) further illustrated the prediction logic for representative samples. Starting from a baseline expected value of E [f(x)] = 0.578, genes such as CFD (value = 9.54, Δ = −0.16), PPARG (value = 6.12, Δ = −0.08), FAAH (value = 7.87, Δ = −0.07), AHR (value = 8.63, Δ = −0.06), and SLC20A2 (value = 8.82, Δ = −0.05) act as major negative contributors, cumulatively lowering the prediction to f(x) = 0.040, leading to the sample’s classification into the control group.

SHAP dependence plots (Figure 5G) capture nonlinear contribution patterns: (1) CFD shows a stable negative SHAP value effect at high expression levels (>8), indicating a consistently negative effect on model output. (2) PRPS1 contributes positively at low expression levels (<6), while its contribution approaches zero at high expression levels. (3) PPARG and AHR exhibit significant negative contributions in low to moderate expression ranges. These findings collectively indicate that the identified core genes influence model predictions through complex, expression-dependent patterns. These bidirectional and nonlinear patterns in model predictions suggest potential functional associations that merit further experimental investigation.

Explanation of SHAP and permutation importance: In our analysis, the mean absolute SHAP values (Figure 5B) identified CFD, PPARG, and PRPS1 as the top contributors to the model’s predictions across all samples. In contrast, permutation importance (Figure 5D) highlighted AHR and SLC20A2 as the most critical for maintaining predictive accuracy. This discrepancy is expected because SHAP measures the average magnitude of each feature’s contribution, while permutation importance quantifies the drop in AUC when a feature is shuffled, reflecting its necessity for the decision boundary. Both rankings are complementary and are reported to provide a complete picture of the model’s logic.

### 2.8. Molecular Docking Confirms TPhP–Core Target Interactions

To explore the potential binding interactions between TPhP and the ten identified core genes (CFD, PRPS1, AHR, SLC20A1, SLC20A2, FAAH, PDE10A, PPARG, BBOX1, and CA2), molecular docking was performed as a computational prediction. According to established criteria in molecular docking studies, binding energy < 0 kcal/mol indicates energetically favorable binding in silico, and <−5.0 kcal/mol is considered good affinity. As shown in Table 3, TPhP showed favorable predicted binding affinities with all ten target proteins, with calculated binding energies ranging from −9.2 kcal/mol to −6.6 kcal/mol. All values were below the commonly used excellent binding threshold of −5.0 kcal/mol, suggesting possible spontaneous interactions in silico.

**Figure 6:**
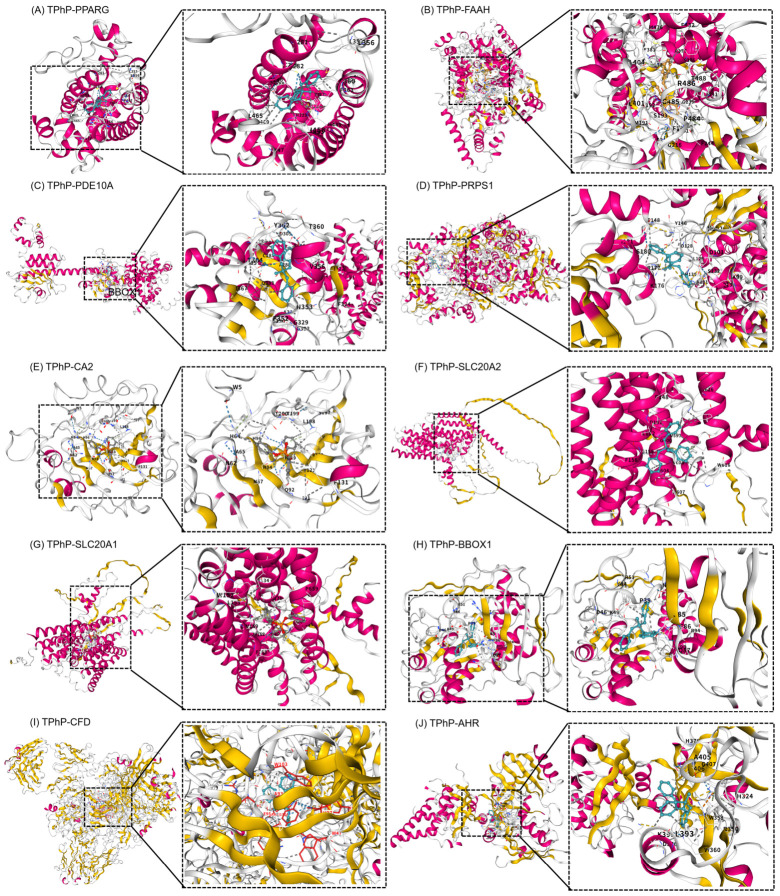
Molecular docking validation of TPhP–core gene interactions. (**A**) TPhP–PPARG. (**B**) TPhP–FAAH. (**C**) TPhP–PDE10A. (**D**) TPhP–PRPS1. (**E**) TPhP–CA2. (**F**) TPhP–SLC20A2. (**G**) TPhP–SLC20A1. (**H**) TPhP–BBOX1. (**I**) TPhP–CFD. (**J**) TPhP–AHR.

The conformational visualization results (Figure 6) showed stable binding modes for each TPhP–protein complex in the docking simulations. Collectively, these computational findings suggest that TPhP may have potential interactions with these ten candidate target proteins. However, as molecular docking is a hypothesis-generating tool, these predicted interactions require experimental validation to confirm actual binding and functional relevance.

## 3. Discussion

In this study, we employed a network toxicology approach to systematically integrate multi-source databases with transcriptomic data to construct a molecular interaction network of triphenyl phosphate (TPhP) in the context of thyroid cancer (TC). Moreover, we combined machine learning and molecular docking techniques to predict and prioritize core targets.

In terms of neurotoxicity, chronic exposure to environmentally relevant doses of TPhP has been demonstrated to substantially decrease the abundance of Lactobacillus in the mouse gut via the gut–brain axis mechanism. It activates the NF-κB signaling pathway in the prefrontal cortex and results in the accumulation of quinolinic acid and glutamate, ultimately inducing behaviors resembling anxiety and depression [17]. During the developmental period, TPhP exposure has been associated with impaired hippocampal synaptogenesis and neurotransmitter transmission, affecting learning and memory abilities [18], and disrupted placental tryptophan metabolism, potentially affecting offspring neurodevelopment [19]. Regarding endocrine disruption, TPhP has been shown to activate the TNF/IL-17/MAPK inflammatory signaling pathways in human thyroid follicular epithelial cells, upregulating mRNA expression of thyroid function-related proteins such as thyroid peroxidase (TPO), thyrotropin receptor (TSHR), and thyroglobulin (TG), thereby potentially promoting thyroid hormone synthesis [20,21]. This inflammatory mechanism is consistent with ADMETlab’s predictions of DILI, skin sensitization, and eye irritation, which may reflect systemic inflammatory effects. Additionally, TPhP and its metabolite diphenyl phosphate (DPhP) have been reported to competitively bind to human thyroid hormone receptors (TR), potentially disrupting normal thyroid signaling [20]. Regarding developmental toxicity, exposure to TPhP resulted in impaired cardiac function in zebrafish embryos, which was manifested as a decreased ejection fraction, reduced heart rate, and increased distance between the sinus venosus and the bulbus arteriosus, accompanied by reduced body length and an enlarged yolk sac area [22]; however, these cardiac toxicities were reported to be reversible post-exposure [23]. At the cellular level, TPhP has been shown to induce apoptosis through excessive production of reactive oxygen species (ROS), changes in mitochondrial membrane potential, and endoplasmic reticulum stress [24,25]. The consistent prediction of mitochondrial membrane potential impairment by both ADMETlab and ProTox-3.0 (Table 1) aligns with these research findings. In mouse spermatocyte cells (GC-2spd), TPhP induced oxidative stress, mitochondrial dysfunction, and DNA damage, triggering apoptosis via a caspase-dependent mitochondrial pathway [26]. Furthermore, TPhP has been associated with hepatotoxicity and lipid and glucose metabolism disorders [27,28] and exhibits immunotoxicity [27] and sex-dependent metabolic disruption [29].

Most of the above-mentioned studies used in vitro or in vivo models with exposure concentrations that may not directly reflect human environmental levels. Regarding the computational predictions, the two platforms (ADMETlab and ProTox-3) showed some discrepancies (e.g., DILI, respiratory toxicity, BBB barrier), but consistently predicted endocrine-related endpoints (ER, AhR, and MMP). This consistency, together with experimental evidence of thyroid hormone disruption and inflammatory activation [20,21], suggests a potential link between TPhP exposure and thyroid cancer risk that may involve endocrine-related pathways. Therefore, TPhP may be associated with thyroid dysfunction and carcinogenesis through these pathways, warranting further investigation. Future studies should include long-term animal studies, human biomonitoring, and experimental validation to confirm the hypothesized links.

We used differential expression analysis, WGCNA, and TPhP target prediction to identify 11 candidate genes overlapping between thyroid cancer signatures and predicted TPhP targets. Further systematic screening of 127 machine learning models selected 10 core genes (CFD, PRPS1, AHR, SLC20A1, SLC20A2, FAAH, PDE10A, PPARG, BBOX1, and CA2), with a combined model AUC of 0.986 on an independent validation set. SHAP analysis revealed the contribution patterns of these genes to the model’s predictions and described how the model uses gene expression levels for classification rather than direct biological regulation. Additionally, we performed molecular docking as a computational prediction to explore potential binding interactions between TPhP and the ten core proteins. The results showed that TPhP exhibited favorable predicted binding affinities with all targets, with all binding energies below the commonly used excellent threshold (Table 3), and stable binding modes were observed for each complex (Figure 6).

CA2 (carbonic anhydrase II) plays a crucial role in maintaining intracellular pH homeostasis and ion transport [30]. As an important member of the zinc metalloenzyme family, CA2 catalyzes the reversible hydration of carbon dioxide, key to maintaining intracellular pH balance, ion transport, and metabolic regulation [31]. In thyroid tissue, CA2 expression levels directly affect the transmembrane transport efficiency of iodide, thereby regulating thyroid hormone synthesis rates [32]. Previous studies have shown that environmental chemicals can disrupt CA2 activity, leading to thyroid hormone homeostasis disorders [33]. Notably, various endocrine-disrupting chemicals have been shown to affect thyroid hormone synthesis and metabolism by interfering with the hypothalamic–pituitary–thyroid (HPT) axis [34]. A recent in vitro study further revealed that TPhP exposure can significantly activate the TNF/IL-17/MAPK inflammatory signaling pathway in human thyroid follicular epithelial Nthy-ori 3-1 cells, upregulating mRNA expression of TPO, TSHR, and TG, thereby promoting thyroid hormone synthesis [20]. Additionally, TPhP and its metabolite DPhP can enhance the binding of thyroxine (T4) to human transthyretin (hTTR) through allosteric regulation, potentially interfering with normal thyroid hormone transport and distribution in the body [21]. Epidemiological studies have also suggested significant associations between organophosphate flame-retardant exposures and thyroid dysfunction, with TPhP metabolite DPhP showing a positive correlation with elevated serum total T4 levels [35].

AHR (aryl hydrocarbon receptor) and PPARG (peroxisome proliferator-activated receptor γ), as typical environmental chemical sensors, play critical roles in mediating the endocrine-disrupting effects of exogenous substances [36,37]. In our study, GO enrichment analysis showed significant enrichment of “nuclear receptor activity” and “ligand-activated transcription factor activity,” and molecular docking confirmed direct binding of TPhP with AHR and PPARG (binding energies of −6.6 kcal/mol and −9.2 kcal/mol, respectively). Activation of AHR can induce the expression of downstream metabolic enzymes such as CYP1A1, promoting other metabolic activations [38], while PPARG influences the tumor microenvironment by regulating lipid metabolism and inflammatory responses. Notably, recent research using CRISPR-Cas9 screens found that AHR deletion in BRAFV600E-positive thyroid cancer cells can promote resistance to MAPK inhibitors via activation of the TGF-*β*/SMAD pathway [39], further supporting the vital role of AHR in thyroid cancer. For PPARG, the PAX8-PPARG fusion gene is the most common molecular marker in follicular thyroid carcinoma, present in about 30% of cases [40,41]. Functional studies have confirmed that PPARG agonists can significantly inhibit thyroid cancer growth and induce adipose differentiation of thyroid cancer cells [42]. The strong influence of AHR and PPARG in our model (most critical for model performance, as indicated by SHAP analysis) suggests that nuclear receptor signaling pathways may be important in the context of TPhP exposure and thyroid cancer, warranting further investigation.

SLC20A1 and SLC20A2 encode type III sodium–phosphate co-transporters, which play key roles in maintaining cellular phosphate homeostasis [43]. In our study, SLC20A2 was significantly downregulated (log_2_FC = −1.832), whereas SLC20A1 was mildly upregulated (log_2_FC = 0.525), suggesting phosphate metabolism reprogramming in thyroid cancer. KEGG enrichment analysis showed significant enrichment of phosphate metabolism-related pathways, such as purine metabolism and the pentose phosphate pathway, indicating that TPhP may be associated with malignant transformation of thyroid cells by disrupting phosphate metabolism reprogramming. PRPS1, a key rate-limiting enzyme in purine metabolism, directly affects nucleotide synthesis and cellular proliferation with its abnormal expression. Our study showed that PRPS1 is significantly downregulated in thyroid cancer tissues (log_2_FC = −1.838), and SHAP analysis indicated that its low expression pushes predictions towards the disease group, with high expression contributing nearly zero, suggesting a nonlinear dependence pattern possibly related to its physiological functional threshold effect. Notably, in vivo studies have shown that TPhP exposure significantly alters the expression of genes related to DNA damage repair pathways, induces DNA oxidative damage (marked by increased 8-OHdG levels), and activates the caspase-dependent apoptosis pathway [44]. This finding aligns well with the enrichment results of phosphate metabolism-related pathways in our study, suggesting that TPhP may promote genetic damage and malignant transformation of thyroid cells by disrupting the balance of nucleotide metabolism and DNA repair.

BBOX1 (γ-butyrobetaine hydroxylase) is the rate-limiting enzyme in carnitine biosynthesis, crucial for mitochondrial fatty acid *β*-oxidation [45]. Recent studies revealed that BBOX1 promotes tumor metastasis by increasing carnitine synthesis and fatty acid oxidation [46]. However, our study found that BBOX1 was significantly downregulated in thyroid cancer tissues (log_2_FC = −1.812), with immunohistochemistry data showing weak to moderate cytoplasmic positive expression in thyroid cancer tissues [47]. The downregulation of BBOX1 may result in reduced carnitine synthesis and fatty acid oxidation capacity, prompting tumor cells to shift to alternative metabolic pathways such as glycolysis to meet energy demands.

CFD, FAAH, and PDE10A are involved in regulating the complement system, endogenous cannabinoid system, and cyclic nucleotide signaling, respectively [48,49,50]. Our study found that CFD is significantly downregulated in thyroid cancer tissues (log_2_FC = −1.848). The downregulation of CFD might lead to impaired alternative pathway activation, weakening the innate immune clearance of tumor cells [51]. The abnormal expression of FAAH (log_2_FC = −1.828) and PDE10A (log_2_FC = −1.818) can affect intracellular levels of second messengers such as cAMP and cGMP, thereby regulating cell proliferation, apoptosis, and inflammatory responses [50,52]. The endogenous cannabinoid system plays an essential role in thyroid function regulation, and abnormal FAAH expression can influence cellular cannabinoid levels, thereby regulating the function of the hypothalamic–pituitary–thyroid axis [53]. A recent in vitro study confirmed that TPhP can activate the TNF/IL-17/MAPK inflammatory signaling pathway in Nthy-ori 3-1 human thyroid follicular epithelial cells, promoting thyroid hormone synthesis [20]. This pathway is directly linked to the PPARG-mediated inflammatory response regulatory module identified in our study, suggesting that activation of inflammatory pathways is a crucial step in TPhP-induced thyroid dysfunction.

The synergistic effects of these core genes reveal a complex network where TPhP drives malignant transformation of thyroid cells by interfering with multiple pathways, including nuclear receptor signaling, phosphate metabolism reprogramming, fatty acid oxidation, inflammation, and ion homeostasis. Importantly, this network is not a simple linear pathway accumulation but forms a complex cross-regulation through key hubs.

Inevitably, this study has several limitations. Firstly, network toxicology analysis is based on computational predictions and public databases; functional validation of core targets requires further confirmation through in vitro and in vivo experiments. Secondly, while molecular docking revealed the binding potential of TPhP with target proteins, it did not account for the metabolic transformation processes of TPhP in vivo (such as activation mediated by the cytochrome P450 enzyme system). Additionally, the transcriptomic data in this study were derived from thyroid tissue samples (cancer vs. normal) and do not contain TPhP-exposed samples; therefore, the identified genes may reflect general thyroid cancer biology rather than TPhP-specific effects, and we could not assess the dose–effect relationship of TPhP exposure or the impact of exposure windows. To further clarify the linkage between real-world exposure and our computational predictions, we examined available human biomonitoring data for TPhP.

Studies have reported urinary concentrations of the TPhP metabolite diphenyl phosphate (DPHP) in various populations, e.g., a geometric mean of 8.01 ng/mL in Hanoi residents [54] and a creatinine-adjusted geometric mean of 1060 pg/mL in the U.S. NHANES 2013–2014 survey [55]. Estimated daily intakes via dust ingestion have been modeled globally [56]. However, the transcriptomic datasets used in the present study do not contain individual-level TPhP exposure measurements, and we did not perform dose-response modeling. Consequently, our mechanistic predictions are not calibrated against actual exposure concentrations and should be interpreted as hypothesis-generating. The relationship between real-world exposure levels and the predicted molecular mechanisms necessitates further exploration in future studies that incorporate exposure-relevant doses. Lastly, although the machine learning model shows high predictive performance in our computational setting, its clinical translatability requires validation through prospective cohort studies. Therefore, future research should utilize various in vitro models (such as thyroid follicular epithelial cell lines) and animal models to further validate the biological functions of core targets and explore dose–effect and time–effect relationships of TPhP exposure, thereby enhancing the reliability and applicability of the study findings.

The computational analysis of potential TPhP-associated thyroid toxicity mechanisms in this study may have significant public health implications. Future research should focus on: (1) developing biomarkers based on the identified core candidate targets for health risk assessment of TPhP exposure; (2) establishing risk assessment models integrating TPhP exposure levels and individual genetic susceptibility (e.g., BRAF mutation status, PAX8-PPARG fusion status); (3) exploring the spatiotemporal regulatory networks of epigenetic modifications and metabolic reprogramming in TPhP-associated thyroid cancer, providing a theoretical basis for the development of targeted intervention strategies.

## 4. Materials and Methods

**Figure 7:**
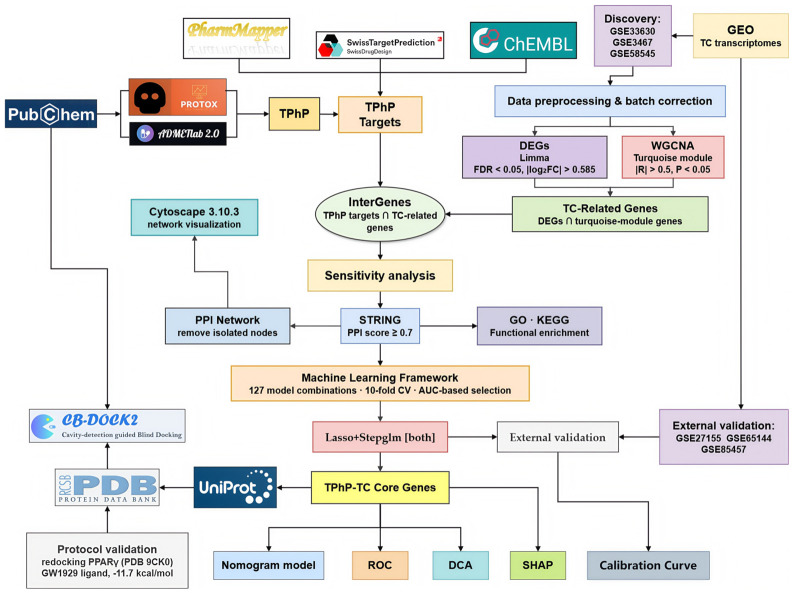
Research flow chart.

This study integrated network toxicology, machine learning benchmarking, and molecular docking to systematically investigate the molecular mechanisms underlying triphenyl phosphate (TPhP)-induced thyroid cancer (TC). The overall research workflow is illustrated in Figure 7.

### 4.1. Toxicity Profiling of TPhP

Chemical structure information for TPhP was retrieved from the PubChem database (https://pubchem.ncbi.nlm.nih.gov/, accessed on 7 August 2025). Comprehensive toxicity assessments and ADMET (Absorption, Distribution, Metabolism, Excretion, and Toxicity) profiles were conducted using the ADMETLAB 3.0 platform (https://admetmesh.scbdd.com/, accessed on 10 August 2025) and the ProTox-3 database (https://tox.charite.de/, accessed on 10 August 2025) to evaluate the environmental and biological safety of the compound.

### 4.2. Retrieval of TPhP-Associated Targets

To identify potential human target genes for TPhP, we integrated three complementary prediction strategies: (1) ligand–receptor interaction data from the ChEMBL database (https://www.ebi.ac.uk/chembl/, accessed on 7 August 2025); (2) chemogenomics-based target prediction via SwissTargetPrediction (http://swisstargetprediction.ch/, accessed on 10 August 2025); (3) 3D pharmacophore matching using PharmMapper (https://lilab-ecust.cn/pharmmapper/index.html, accessed on 10 August 2025). We retained all human targets from ChEMBL and SwissTargetPrediction (probability > 0) and only those with a normalized fit score > 0.6 from PharmMapper. After merging results from these sources and removing redundancies, a comprehensive set of TPhP-associated human targets was established.

### 4.3. Identification of TC-Related Genes

#### 4.3.1. GEO Data Acquisition and Differential Expression Analysis

We collected six TC transcriptomic datasets from the NCBI Gene Expression Omnibus (GEO) database, including GSE33630, GSE3467, GSE58545, GSE27155, GSE65144, and GSE85457. GSE33630, GSE3467, and GSE58545 were used as the discovery cohort, whereas GSE27155, GSE65144, and GSE85457 served as the validation cohort. These datasets contain gene expression profiles of human thyroid cancer tissues and adjacent normal thyroid tissues. To control for batch effects, we applied a multistep normalization strategy. First, surrogate variable analysis (SVA) was used to model and correct potential confounding factors in the discovery cohort. We then used the ComBat method under a parametric empirical Bayes framework to further remove residual batch effects. Principal component analysis (PCA) after correction showed markedly improved clustering of samples from different batches in low-dimensional space, which confirmed the effectiveness of data integration.

#### 4.3.2. Differential Gene Expression Analysis

Using the Limma package, we performed differential expression analysis and defined differentially expressed genes (DEGs) as those with an FDR-adjusted *p* value < 0.05 and |log_2_FC| > 0.585 (corresponding to a 1.5-fold change). These thresholds are commonly adopted in transcriptomic studies to balance biological relevance with false discovery control. The results were visualized using the ggplot2 package.

#### 4.3.3. Weighted Gene Co-Expression Network Analysis (WGCNA)

We constructed a scale-free co-expression network using the WGCNA package. Sample quality was first assessed by hierarchical clustering, and outliers were removed. The optimal soft-thresholding power was then determined using the dynamic tree-cutting method, with a scale-free topology fit index of R^2^ > 0.85 as the selection criterion. Gene modules were identified by hierarchical clustering based on the topological overlap matrix, with a minimum module size of 30 and a threshold of 0.25 for merging similar modules. Module–trait associations were evaluated by correlating module eigengenes with phenotypic traits. A Pearson correlation coefficient of |R| > 0.5 and *p* < 0.05 were considered significant. The turquoise module showed the strongest negative correlation with the cancer trait (Pearson R = −0.87, *p* = 3.6 × 10^−54^). We therefore selected all genes from the turquoise module (*n* = 1064) for subsequent analysis. We did not apply additional filtering by module membership (kME) in order to retain a broad set of co-expressed genes.

#### 4.3.4. Integration of DEGs and WGCNA Module Genes

To obtain a set of thyroid cancer (TC)-related candidate genes, we integrated the DEGs identified in Section 4.3.2 with the turquoise module genes identified in Section 4.3.3. This set represents TC-associated genes for subsequent overlap analysis with TPhP targets. The intersection results were visualized using Venn diagrams.

### 4.4. Overlap Analysis of TPhP Targets and TC-Related Genes

#### 4.4.1. Intersection Analysis with TPhP Targets

To identify candidate genes that may link TPhP to thyroid cancer, we intersected the TC-associated genes (obtained in Section 4.3.4) with the TPhP-predicted targets (Section 4.2). The overlapping TPhP–TC targets were visualized through Venn diagrams and saved for subsequent analyses.

#### 4.4.2. Sensitivity Evaluation of DEG Thresholds

To assess the robustness of our gene selection to the choice of DEGs thresholds, we repeated the DEG identification (Section 4.3.2) using three alternative cutoffs: lenient (|log_2_FC| > 0.3, FDR < 0.1), original (|log_2_FC| > 0.585, FDR < 0.05), and stringent (|log_2_FC| > 1.0, FDR < 0.01). For each threshold set, we performed the same integration with turquoise module genes (Section 4.3.4) and the final intersection with TPhP targets (Section 4.4.1).

#### 4.4.3. PPI Network Construction

We submitted the overlapping genes obtained from the original thresholds (|log_2_FC| > 0.585, FDR < 0.05) to the STRING database (https://string-db.org/, accessed on 17 September 2025) for protein–protein interaction (PPI) analysis, using a confidence score threshold of ≥0.7. We excluded isolated nodes to retain biologically relevant interactions. We visualized the resulting PPI network using Cytoscape 3.10.3 (https://cytoscape.org/, accessed on 19 September 2025).

### 4.5. Functional Enrichment Analysis of TPhP–TC Core Targets

Functional enrichment analysis was conducted on intersecting genes (InterGenes) using the “clusterProfiler” R package to perform Gene Ontology (GO) and Kyoto Encyclopedia of Genes and Genomes (KEGG) pathway analysis, with a significance threshold set at *p* < 0.05. Visualization of the functional enrichment results was achieved through the use of the “ggplot2”, “circlize”, and “ComplexHeatmap” R packages. This hypothesis-generating analysis explores potential functional pathways that may associate TPhP-related targets with thyroid cancer.

### 4.6. Machine Learning-Based Identification and Validation of Core Targets

Data source and cohort partition: Six thyroid cancer transcriptomic datasets were obtained from GEO. To enable rigorous external validation and prevent data leakage, we divided them into a discovery cohort (GSE33630, GSE3467, GSE58545; merged and batch-corrected as a single matrix using ComBat exclusively within these three datasets) and three independent validation datasets (GSE27155, GSE65144, GSE85457; each kept as original, without batch correction). The discovery cohort was used for all feature selection, model training, and internal cross-validation. The validation datasets were kept completely untouched until the final model was locked.

Preprocessing: Raw expression data in the discovery cohort were Z-score standardized (mean = 0, SD = 1) using parameters estimated only from the discovery cohort. Gaussian noise (mean = 0, SD = 0.01) was added to improve robustness. No batch correction was applied to the validation datasets; they were independently standardized using their own means and SDs to avoid any information leakage.

Feature selection and model training: We developed an integrated framework combining 127 predictive models. The algorithms included: Lasso regression (glmnet, alpha = 1), Ridge regression (glmnet, alpha = 0), Elastic Net (glmnet, alpha between 0–1), Random Forest (randomForestSRC, ntree = 1000, nodesize = 5), Gradient Boosting Machine (GBM, gbm, n.trees = 10,000, interaction.depth = 3, shrinkage = 0.001, cv.folds = 10), Support Vector Machines (e1071), Naive Bayes (e1071), Partial Least Squares Generalized Linear Models (plsRglm), Bayesian Additive Regression Trees (BART), and others. For combination methods (e.g., “Lasso + SVM” or “glmBoost + Ridge”), the first algorithm was used for feature selection, followed by the second for modeling. Stepwise regression (forward, backward, bidirectional) was also employed.

All steps were performed exclusively within the discovery cohort using 10-fold cross-validation (CV). A two-stage feature selection strategy was applied: First, for each method, significant variables were automatically selected from the training fold using the corresponding algorithm (e.g., Lasso, Random Forest, GBM) via 10-fold CV, with a minimum variable threshold of 2; if fewer were selected, t-test *p*-values (calculated on the training fold only) were used to supplement the feature set to reach the threshold. Second, classification models were constructed using the selected feature sets. All methods were executed via a custom function RunML, with key parameters based on the literature and experience (e.g., Lasso alpha = 1, Ridge alpha = 0, Elastic Net alpha between 0 and 1, Random Forest ntree = 1000, GBM n.trees = 10,000).

Model development and internal cross-validation: A total of 127 model combinations were evaluated using 10-fold cross-validation exclusively within the discovery cohort. For each model, feature selection and hyperparameter tuning were performed inside each cross-validation fold using only the training fold. The performance metric was the area under the ROC curve (AUC). The model with the highest mean cross-validation AUC was selected as the final model. Its cross-validation variability (mean AUC, standard deviation, and range) was recorded. After selection, the final model was retrained on the full discovery cohort and then locked.

External validation: The locked final model was evaluated once on each of the three independent validation datasets (GSE27155, GSE65144, GSE85457). For each dataset, we report the AUC with 95% confidence intervals (bootstrap, 2000 resamples), sensitivity, specificity, and Brier score (calibration). Calibration curves were plotted to assess agreement between predicted and observed probabilities (Figure 4B). Decision curve analysis (DCA) and nomogram were performed in the discovery cohort to evaluate clinical net benefit. A heatmap of model performance was generated using the ComplexHeatmap package to visualize the AUCs of different models on the validation datasets (Figure 4A).

### 4.7. Model Interpretation (SHAP)

To enhance model interpretability and overcome the “black-box” limitation of machine learning algorithms, this study adopted the SHAP (SHapley Additive exPlanations) method. Based on the core gene set screened through the multiple machine learning models, SHAP quantified the marginal contribution and effect direction of each feature gene on predictive outcomes from a global perspective by calculating feature contribution values for each sample. This analysis clarified the importance ranking of the core genes and revealed the logical associations between gene expression levels and disease state predictions. It provided evidence not only of statistical significance and predictive accuracy but also of decision-making logic, supporting subsequent molecular docking validation. In addition, we computed permutation importance (by shuffling each feature and measuring the drop in AUC) to provide a complementary view of feature necessity. Both metrics are reported together in the Section 2.7.

### 4.8. Molecular Docking Analysis

To explore the potential binding interactions between TPhP and the ten identified core proteins, we retrieved the SDF structure file of TPhP from PubChem (https://pubchem.ncbi.nlm.nih.gov/, accessed on 7 August 2025) and the three-dimensional protein structures of the core targets from UniProt (https://www.uniprot.org/uniprotkb, accessed on 22 March 2026). Molecular docking was then performed using CB-Dock2 (https://cadd.labshare.cn/cb-dock2/index.php, version 2.0, accessed on 24 March 2026) [57]. The protein and ligand structures were directly submitted to the server, which automatically performed structure preparation (including repairing missing atoms, adding hydrogen atoms, and removing water molecules and other heteroatoms) and cavity detection. Docking calculations were carried out using AutoDock Vina (version 1.2.0) as the embedded engine. Binding energy (kcal/mol) was used to evaluate ligand–receptor interactions; a binding energy < 0 kcal/mol indicates energetically favorable binding in silico, and <−5 kcal/mol is considered good affinity. To validate the reliability of the docking protocol, we performed a redocking experiment using PPARγ (PDB ID: 9CK0) and its co-crystallized ligand GW1929 (EDK). The ligand was extracted from the crystal structure and re-docked into the same binding pocket using the same CB-Dock2 parameters. The redocking successfully reproduced the native binding mode with a strong binding energy of −11.7 kcal/mol, confirming that our docking settings are appropriate and the predictions are reliable. All docking results are reported as computational predictions and require experimental validation (e.g., qPCR, Western blotting, or functional assays) to confirm actual binding and functional relevance.

## Figures and Tables

**Table 1:** Comparison of key toxicity endpoints predicted by ADMETlab 2.0 and ProTox-3.0 for TPhP.

Toxicity Endpoint/Pathway	ADMETlab 2.0 (Probability/Call)	ProTox-3.0 (Prediction/Probability)	Agreement
DILI (hepatotoxicity)	0.969 (Active)	Inactive (0.74)	Disagree
Skin sensitization	0.974 (Active)	Not reported	–
Eye irritation	0.988 (Active)	Not reported	–
Respiratory toxicity	0.711 (Active)	Inactive (0.98)	Disagree
hERG blocker	0.761 (Active)	Cardiotoxicity inactive (0.69)	Partially agree
BBB barrier	BBB penetration 0.376	Active (0.82)	Disagree
Ecotoxicity	LC50 values provided	Active (0.73)	–
AhR (aryl hydrocarbon receptor)	0.933 (Active)	Active (0.61)	Agree
ER (estrogen receptor)	0.907 (Active)	Active (0.99)	Agree
Mitochondrial membrane potential	0.801 (Active)	Active (0.99)	Agree
AR/AR-LBD	<0.02 (Inactive)	>0.99 (Inactive)	Agree
PPAR-γ 0.005	0.005 (Inactive)	1.0 (Inactive)	Agree

Note: “–“ indicates that the endpoint was not directly comparable between tools. Different tools use different probability thresholds for active/inactive calls; direct comparison is only indicative.

**Table 2:** External validation performance of the final model on three independent datasets.

Dataset	AUC (95% CI)	Sensitivity	Specificity	Brier Score
GSE27155	0.997 (0.984–1.000)	0.989	1.000	0.202
GSE65144	0.994 (0.962–1.000)	0.833	0.923	0.094
GSE85457	1.000 (1.000–1.000)	1.000	1.000	0.032

**Table 3:** Binding energy of ligands with receptors.

Ligand	Receptor	Binding Energy (kcal/mol)
TPhP	PPARG	−9.2
TPhP	FAAH	−8.6
TPhP	PDE10A	−8.5
TPhP	PRPS1	−8.0
TPhP	CA2	−7.9
TPhP	SLC20A2	−7.3
TPhP	SLC20A1	−7.1
TPhP	BBOX1	−7.1
TPhP	CFD	−7.1
TPhP	AHR	−6.6

## Data Availability

The raw data, query snapshots, and all custom scripts generated during this study are deposited in a public repository to ensure full reproducibility. The datasets and code are available at GitHub (https://github.com/pyll666/tphp-thyroid-cancer-ml-docking, accessed on 24 March 2026) and are archived on Zenodo with the DOI https://doi.org/10.5281/zenodo.21044226. The repository includes the original input data, intermediate filtering results, and all analysis scripts (R). All files are shared under a Creative Commons Attribution 4.0 International (CC BY 4.0) license.

## Supplementary Materials

The following supporting information can be downloaded at https://www.mdpi.com/article/10.3390/ijms27136018/s1.

## Abbreviations

The following abbreviations are used in this manuscript:

TPhP	Triphenyl phosphate
TC	Thyroid cancer
EDCs	Endocrine-disrupting chemicals
SHAP	SHapley Additive exPlanation
DEGs	Differentially expressed genes
GEO	Gene Expression Omnibus
WGCNA	Weighted Gene Co-Expression Network Analysis
GO	Gene Ontology
KEGG	Kyoto Encyclopedia of Genes and Genomes
AUC	Area Under the Curve
ROC	Receiver Operating Characteristic
DCA	Decision Curve Analysis
PPI	Protein–Protein Interaction
SDF	Structure Data File
PCA	Principal Component Analysis
CI	Confidence Interval
AHR	Aryl Hydrocarbon Receptor
PPARG	Peroxisome Proliferator-Activated Receptor γ
CA2	Carbonic Anhydrase II
SLC20A2	Solute Carrier Family 20 Member 2
CFD	Complement Factor D
PRPS1	Phosphoribosyl Pyrophosphate Synthetase 1
FAAH	Fatty Acid Amide Hydrolase
PDE10A	Phosphodiesterase 10A
BBOX1	γ-butyrobetaine Hydroxylase 1
SLC20A1	Solute Carrier Family 20 Member 1
CYP1A1	Cytochrome P450 1A1
DPhP	Diphenyl Phosphate
TNF	Tumor Necrosis Factor
IL-17	Interleukin-17
MAPK	Mitogen-Activated Protein Kinase
TPO	Thyroid Peroxidase
TSHR	Thyrotropin Receptor
TG	Thyroglobulin
HPT	Hypothalamic–Pituitary–Thyroid
T4	Thyroxine
hTTR	Human Transthyretin
BRAF	B-Raf Proto-oncogene
TGF-*β*	Transforming Growth Factor-beta
SMAD	Mothers against Decapentaplegic Homolog
PAX8	Paired Box Gene 8
8-OHdG	8-hydroxy-2′-deoxyguanosine
cAMP	Cyclic Adenosine Monophosphate
cGMP	Cyclic Guanosine Monophosphate
RF	Random Forest
SVM	Support Vector Machine
XGB	XGBoost
GBM	Gradient Boosting Machine
KNN	K-Nearest Neighbor
Lasso	Least Absolute Shrinkage and Selection Operator
Ridge	Ridge Regression
