# Supplementary material for: Potential Molecular Associations Between Triphenyl Phosphate Exposure and Thyroid Cancer: Integration of Network Toxicology and Machine Learning for Core Target Identification with Molecular Docking
Source: Int J Mol Sci. 2026 Jul 4;27(13):6018. doi: 10.3390/ijms27136018 (PMC13362510; doi:10.3390/ijms27136018)
Supplement: Supplementary file 1 [file ijms-27-06018-s001.zip › Supplementary Material S2.pdf]

Oral toxicity prediction results for input compound

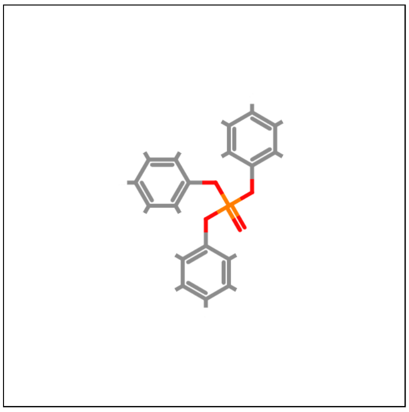

Predicted LD50: 1320mg/kg

Predicted Toxicity Class: 4

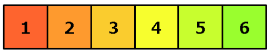

Average similarity: 100%

Prediction accuracy: 100%

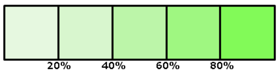

Name	Triphenyl Phosphate
Molweight	326.28
Number of hydrogen bond acceptors	4
Number of hydrogen bond donors	0
Number of atoms	23
Number of bonds	25
Number of rotable bonds	6
Molecular refractivity	88.82
Topological Polar Surface Area	54.57
octanol/water partition coefficient(logP)	5.33

Toxicity Model Report

Copy Excel CSV PDF

Classification	Target	Shorthand	Prediction	Probability
Organ toxicity	Hepatotoxicity	dili	Inactive	0.74
Organ toxicity	Neurotoxicity	neuro	Inactive	0.85
Organ toxicity	Nephrotoxicity	nephro	Inactive	0.56
Organ toxicity	Respiratory toxicity	respi	Inactive	0.98
Organ toxicity	Cardiotoxicity	cardio	Inactive	0.69
Toxicity end points	Carcinogenicity	carcino	Inactive	0.76
Toxicity end points	Immunotoxicity	immuno	Inactive	0.99
Toxicity end points	Mutagenicity	mutagen	Inactive	0.96
Toxicity end points	Cytotoxicity	cyto	Inactive	0.83
Toxicity end points	BBB-barrier	bbb	**Active**	0.82
Toxicity end points	Ecotoxicity	eco	**Active**	0.73
Toxicity end points	Clinical toxicity	clinical	Inactive	0.71
Toxicity end points	Nutritional toxicity	nutri	Inactive	0.54
Tox21-Nuclear receptor signalling pathways	Aryl hydrocarbon Receptor (AhR)	nr_ahr	**Active**	0.61
Tox21-Nuclear receptor signalling pathways	Androgen Receptor (AR)	nr_ar	Inactive	0.99
Tox21-Nuclear receptor signalling pathways	Androgen Receptor Ligand Binding Domain (AR-LBD)	nr_ar_lbd	Inactive	1.0
Tox21-Nuclear receptor signalling pathways	Aromatase	nr_aromatase	Inactive	0.99
Tox21-Nuclear receptor signalling pathways	Estrogen Receptor Alpha (ER)	nr_er	**Active**	0.99
Tox21-Nuclear receptor signalling pathways	Estrogen Receptor Ligand Binding Domain (ER-LBD)	nr_er_lbd	Inactive	0.99
Tox21-Nuclear receptor signalling pathways	Peroxisome Proliferator Activated Receptor Gamma (PPAR-Gamma)	nr_ppar_gamma	Inactive	1.0
Tox21-Stress response pathways	Nuclear factor (erythroid-derived 2)-like 2/antioxidant responsive element (nrf2/ARE)	sr_are	Inactive	0.99
Tox21-Stress response pathways	Heat shock factor response element (HSE)	sr_hse	Inactive	0.99
Tox21-Stress response pathways	Mitochondrial Membrane Potential (MMP)	sr_mmp	**Active**	0.99
Tox21-Stress response pathways	Phosphoprotein (Tumor Supressor) p53	sr_p53	Inactive	0.99
Tox21-Stress response pathways	ATPase family AAA domain-containing protein 5 (ATAD5)	sr_atad5	Inactive	0.99
Molecular Initiating Events	Thyroid hormone receptor alpha (THRα)	mie_thr_alpha	Inactive	0.90
Molecular Initiating Events	Thyroid hormone receptor beta (THRβ)	mie_thr_beta	Inactive	0.78
Molecular Initiating Events	Transtyretrin (TTR)	mie_ttr	Inactive	0.97
Molecular Initiating Events	Ryanodine receptor (RYR)	mie_ryr	Inactive	0.98
Molecular Initiating Events	GABA receptor (GABAR)	mie_gabar	Inactive	0.96
Molecular Initiating Events	Glutamate N-methyl-D-aspartate receptor (NMDAR)	mie_nmdar	Inactive	0.92
Molecular Initiating Events	alpha-amino-3-hydroxy-5-methyl-4-isoxazolepropionate receptor (AMPAR)	mie_ampar	Inactive	0.97
Molecular Initiating Events	Kainate receptor (KAR)	mie_kar	Inactive	0.99
Molecular Initiating Events	Acetylcholinesterase (AChE)	mie_ache	Inactive	0.74
Molecular Initiating Events	Constitutive androstane receptor (CAR)	mie_car	Inactive	0.98
Molecular Initiating Events	Pregnane X receptor (PXR)	mie_pxr	Inactive	0.92

Classification	Target	Shorthand	Prediction	Probability
Molecular Initiating Events	NADH-quinone oxidoreductase (NADHOX)	mie_nadhox	Inactive	0.97
Molecular Initiating Events	Voltage gated sodium channel (VGSC)	mie_vgsc	Inactive	0.95
Molecular Initiating Events	Na+/I- symporter (NIS)	mie_nis	Inactive	0.98
Metabolism	Cytochrome CYP1A2	CYP1A2	Inactive	0.65
Metabolism	Cytochrome CYP2C19	CYP2C19	Inactive	0.66
Metabolism	Cytochrome CYP2C9	CYP2C9	**Active**	0.60
Metabolism	Cytochrome CYP2D6	CYP2D6	Inactive	0.79
Metabolism	Cytochrome CYP3A4	CYP3A4	**Active**	0.65
Metabolism	Cytochrome CYP2E1	CYP2E1	Inactive	0.99

Toxicity targets

Possible binding to toxicity targets is shown below. For more information on the targets, please click on the individual abbreviations.

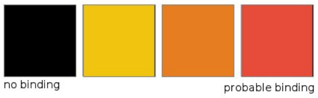

AA2AR	ADRB2	ANDR	AOFA	CRFR1	DRD3	ESR1	ESR2	GCR	HRH1	NR1I2	OPRK	OPRM	PDE4D	PGH1	PRGR
